# TRIMming SIV Transmission between Species

**DOI:** 10.1371/journal.pbio.1000463

**Published:** 2010-08-24

**Authors:** Caitlin Sedwick

**Affiliations:** Freelance Science Writer, San Diego, California, United States of America

**Figure pbio-1000463-g001:**
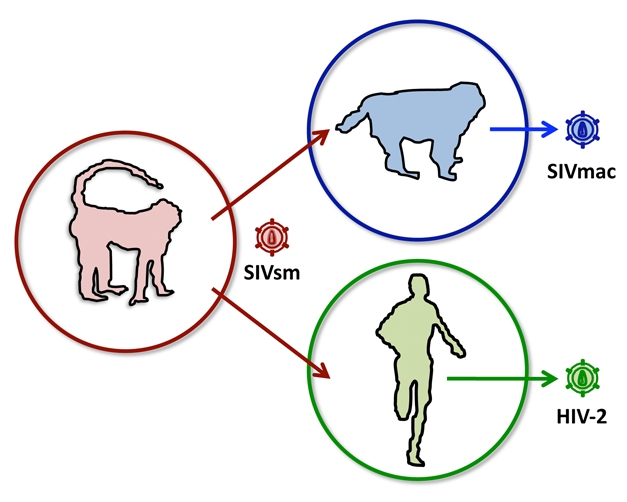
The primate immunodeficiency viruses have a history of jumping species and adapting to new hosts, emerging as “new” species of virus. Host genes like TRIM5 may be key players in this process, influencing the probability of transmission and forcing adaptation of the emerging virus.

[Fig pbio-1000463-g001]Human immunodeficiency virus (HIV), the virus that causes AIDS, did not originate in humans. Instead, the most common variant, HIV1, is thought to have originated as a simian immunodeficiency virus (SIV) in apes and to have jumped the species barrier from apes to humans sometime in the first half of the 20th century. Similarly, HIV2—a related but less virulent virus—arrived in humans after jumping from a different primate species, the sooty mangabey. SIVs have also jumped between different primate populations, both in the wild and in animals kept in captivity for research purposes. But, just how SIV was able to gain a toehold in new, genetically distinct species has been something of a puzzle; laboratory experiments suggest that there exist several host genes that should, in theory, pose a formidable barrier to interspecies viral transmission. The genes encode what are known collectively as restriction factors, and they are thought to have evolved specifically to prevent movement of viruses between species. Understanding how viruses overcome these barriers to transmission is important for designing both vaccines and treatments. In this issue of *PLoS Biology*, Andrea Kirmaier, Welkin Johnson, and colleagues provide new insight about the impact the macaque gene *TRIM5* makes on cross-species transmission of SIV.

In the 1970s, before SIV was recognized as a potential cross-species contagion, a group of captive rhesus macaques in the United States was accidentally exposed to SIV from sooty mangabeys. The sooty mangabey viral strain, called SIVsm, quickly adapted to its new host, becoming a new, genetically distinct strain called SIVmac. It was subsequently recognized that infected macaques develop an immune deficiency syndrome very similar to that observed in HIV-infected humans. For that reason, SIV-infected macaques are now closely studied as an animal model for HIV disease. But how did SIVsm cross the species barrier in the first place?

One obstacle to cross-species viral transmission is posed by the monkeys' *TRIM5* gene, whose protein product, TRIM5α, blocks lentiviruses (the viral family that counts SIV and HIV as members) shortly after they infect a host cell by binding to viral capsid proteins. Johnson's research group had earlier shown that macaques possess several different alleles of the *TRIM5* gene, each of which encodes a slightly different TRIM5α protein. Experiments with cells expressing the different *TRIM5* alleles showed that some of the alleles strongly restrict the replication of certain lentiviruses, while others are quite permissive for replication.

Kirmaier et al. wondered whether, in jumping from sooty mangabeys to macaques, SIV had adapted to the presence of macaque *TRIM5* alleles. They therefore tested to what extent the different macaque *TRIM5* alleles restrict the growth of an SIV strain isolated directly from sooty mangabeys. This experiment showed that most *TRIM5* alleles strongly inhibit the growth of this strain; only one *TRIM5* allele, called TRIM5^Q^, fails to restrict its growth at all. But then the group tested the *TRIM5* alleles against SIVmac, a viral strain that had originated in sooty mangabeys but had been growing in macaque populations for a long time. In this case, they found that none of the *TRIM5* alleles could restrict the growth of the virus.

The cell culture experiments suggest that SIVsm starts out susceptible to most macaque *TRIM5* alleles, but eventually adapts to become resistant to all alleles. Kirmaier and colleagues wondered whether such adaptations also occur in animals. Fortunately, they had access to archived macaque blood and virus samples that other groups had collected during studies with SIV-infected macaques. Retrospective analyses performed on these samples indicated that SIVsm strains replicate best in macaques that have two copies of the TRIM5^Q^ allele. In fact, viral replication levels correlated better with the animals' *TRIM5* genotype than with other genetic factors thought to affect SIV replication. However, these studies also showed that SIV could readily mutate to escape restriction by the other *TRIM5* alleles; within a year, even some of the animals with two restrictive *TRIM5* alleles had readily detectable levels of SIV in their blood.

What kinds of mutations allow SIV to escape restriction by *TRIM5*? Sequencing the capsid region of “escaped” viruses showed novel mutations in the amino acid sequence of macaque-adapted, *TRIM5*-resistant SIV that are not present in sooty mangabey-adapted SIV. When the authors used molecular techniques to revert the escaped virus' capsid sequence back to that found in sooty mangabey-adapted SIV, they found that the virus once again became susceptible to restriction by *TRIM5* alleles (other than TRIM5^Q^) in cell culture.

Collectively, these studies show that *TRIM5* exerts pressure on SIV during the early stages of cross-species viral transmission. This conclusion is interesting from the perspective of viral evolution, but it also has practical implications: designers of studies on SIV-infected macaques should consider whether the SIV they plan to study is already *TRIM5*-adapted before attempting to reach any conclusions about vaccine or treatment efficacy.

**Kirmaier A, Wu F, Newman RM, Hall LR, Morgan JS, et al. (2010)** ***TRIM5*** **Suppresses Cross-Species Transmission of a Primate Immunodeficiency Virus and Selects for Emergence of Resistant Variants in the New Species. doi:10.1371/journal.pbio.1000462**

